# What Drives Indonesian People to Purchase Modest Fashion? The Moderating Role of Religiosity

**DOI:** 10.12688/f1000research.173033.3

**Published:** 2026-06-10

**Authors:** Muhammad Anwar Fathoni, Dienni Ruhjatini Sholihah, Ajeng Septiana Wulansari

**Affiliations:** 1 Islamic Economics, Universitas Pembangunan Nasional Veteran Jakarta, Jakarta, Special Capital Region of Jakarta, Indonesia; 2 Management, Universitas Pembangunan Nasional Veteran Jakarta, Special Capital Region of Jakarta, Indonesia

**Keywords:** Modest fashion, purchase decision, Islamic marketing

## Abstract

**Purpose:**

This study aims to examine how religiosity moderates the influence of attitude and electronic word of mouth (E-WOM) on purchase decisions in Indonesia’s modest fashion market. It seeks to provide empirical evidence on how cognitive, social, and spiritual factors interact in shaping Muslim consumers’ purchasing behavior.

**Design/methodology/approach:**

A quantitative research approach was employed using Structural Equation Modeling (SEM) with data collected from 320 Muslim consumers in Jakarta and surrounding areas. The constructs (attitude, E-WOM, religiosity, and purchase decision) were measured using validated multi-item scales adapted from previous studies.

**Findings:**

The results indicate that both attitude and E-WOM have significant positive effects on purchase decisions, with E-WOM being the most dominant factor. Religiosity exerts a direct positive influence and strengthens the relationship between attitude and purchase decision, but does not moderate the effect of E-WOM. These findings highlight that while religiosity enhances value-driven behavior, digital influence transcends religious intensity.

**Practical implications:**

The study suggests that modest fashion marketers should integrate syariah-compliant values with credible digital engagement strategies to foster consumer trust and loyalty. Balancing faith-based authenticity with modern digital communication can enhance brand relevance in the halal fashion market.

**Originality/value:**

This study extends the Theory of Planned Behavior (TPB) by incorporating religiosity as a moderating factor within the context of Islamic consumer behavior. It contributes to the growing body of Islamic marketing literature by revealing how faith and digital interaction jointly shape purchase decisions in the modest fashion industry.

## 1. Introduction

The modest fashion industry in Indonesia has been experiencing rapid growth and has positioned itself as a major contributor to the global halal economy ( Yuniastuti & Pratama, 2023). As the country with the largest Muslim population in the world, Indonesia offers not only a vast market but also a unique consumer landscape. For Muslim consumers, modest fashion represents more than clothing choices. It reflects religious identity, moral values, and social aspirations ( Furehaug, 2024; Mirza, 2024; Munawaroh et al., 2025). This phenomenon highlights the importance of understanding the factors that drive purchase decisions in the modest fashion sector.

In this study, modest fashion refers to clothing that adheres to Islamic principles of modesty, including covering the body appropriately, avoiding transparency and tightness, and reflecting ethical and cultural values. Beyond its functional aspect, modest fashion also represents a form of identity expression that integrates religious commitment, aesthetic preference, and contemporary lifestyle ( Aruan & Wirdania, 2020; Mirza, 2024). Thus, modest fashion is conceptualized not merely as a dress code, but as a socio-cultural and religious phenomenon embedded in consumer behavior.

Purchase decisions in modest fashion are shaped by both psychological and social factors ( Manzoor et al., 2024; Sholihah et al., 2025). Attitude has often been emphasized as a key predictor of consumer behavior, as it reflects individual evaluations of modest fashion products across cognitive, affective, and conative dimensions ( Sumarliah et al., 2022). However, a favorable attitude may not be sufficient to drive actual decisions, as consumers also rely on information, social validation, and religious justification in their choices ( Kim & Rana, 2025; Naeem & Ozuem, 2021).

At the same time, the rise of digital platforms has made electronic word of mouth (E-WOM) increasingly influential ( Dehyadegari et al., 2025; Matiukaite et al., 2024; Singh et al., 2023). Reviews, recommendations, and consumer-generated content about modest fashion disseminated through social media and online platforms provide powerful signals that can shape perceptions, reduce uncertainty, and strengthen consumers’ confidence in their purchase decisions ( Almashaleh & Fatahi Valilai, 2025).

Another factor that deserves decision is religiosity. In the Islamic context, religiosity serves not only as a spiritual identity but also as a guiding principle for daily behavior. Religious consumers are more likely to ensure that their choices, including those related to modest fashion, align with Islamic values ( Amalanathan & Reddy-Best, 2024; Aruan & Wirdania, 2020). Beyond its direct role, religiosity may moderate the relationship between consumer attitudes, E-WOM, and purchase decisions. It can reinforce or weaken the effects of these variables, depending on the degree of religious commitment ( Akhtar et al., 2023; Dehyadegari et al., 2025; Nickerson et al., 2023).

Although prior studies have examined the role of attitude, religiosity, and word of mouth in halal-related consumption, limited attention has been given to modest fashion in Indonesia. Specifically, there is a lack of research that simultaneously examines the influence of attitude and E-WOM on purchase decisions, while considering religiosity as a moderating factor. This study seeks to fill this gap by investigating the effects of attitude and E-WOM on purchase decisions in modest fashion, and by analyzing the moderating role of religiosity. The findings are expected to contribute to the literature on Islamic marketing and provide practical insights for modest fashion businesses in Indonesia.

While the Theory of Planned Behavior (TPB) has been widely used to explain consumer decision-making, its application in Islamic consumption contexts remains theoretically underdeveloped. TPB emphasizes attitude as a key determinant of behavior; however, it does not explicitly account for external digital influences such as electronic word of mouth (E-WOM) or internal spiritual drivers such as religiosity. This limitation is particularly relevant in the modest fashion context, where consumption is not only driven by rational evaluation but also shaped by social validation and religious commitment. Prior studies have produced inconsistent findings regarding the role of religiosity, some suggest it directly influences purchase decisions, while others indicate it acts as a boundary condition that strengthens or weakens behavioral relationships. Similarly, although E-WOM is recognized as a powerful driver in digital environments, its integration into TPB-based models remains fragmented.

This study focuses on purchase decision as a proxy for actual behavior, rather than purchase intention. While intention reflects a consumer’s willingness to act, purchase decision captures a more concrete behavioral outcome, representing the final stage of the decision-making process. Therefore, this study develops an extended TPB framework by incorporating E-WOM as a form of social influence and religiosity as a moderating variable. This integration provides a more comprehensive explanation of how cognitive (attitude), social (E-WOM), and spiritual (religiosity) factors interact in shaping purchase decisions in the modest fashion market. This represents a conceptual contribution, not merely a contextual extension.

## 2. Literature review and hypotheses development

### 2.1 Attitude and purchase decision

Attitude refers to an individual’s learned predisposition to evaluate an object positively or negatively, reflecting cognitive, affective, and behavioral tendencies. It reflects a psychological orientation that develops through experience, social interaction, and personal values, influencing how a person thinks, feels, and behaves toward something. In essence, attitude embodies the internal evaluation that bridges one’s cognition, emotion, and behavior, forming the basis for how individuals interpret and react to their environment.

In the context of consumer behavior, attitude plays a crucial role in determining how consumers perceive products and make purchasing decisions. It represents an underlying mindset that guides their evaluation of product attributes, brand image, and overall satisfaction. A positive attitude generally leads to greater willingness to purchase, recommend, or remain loyal to a brand, while a negative attitude may result in avoidance or rejection.

In the case of modest fashion, for example, attitude influences how Muslim consumers perceive clothing that aligns with Islamic values of decency and modesty. When consumers develop a positive attitude toward modest fashion, they are more likely to engage in actual purchase behavior. Thus, attitude does not stand in isolation; it is shaped by various internal and external factors, including personal beliefs, cultural context, social influence, and religiosity.

Within the Theory of Planned Behavior ( Ajzen, 1991), attitude plays a central role in shaping both intention and actual behavior. In the context of modest fashion, consumer attitudes are formed through cognitive beliefs (e.g., knowledge of syariah compliance and quality), affective evaluations (e.g., feelings of pride or identity expression), and conative tendencies (e.g., readiness to act).

In this study, attitude toward modest fashion is conceptualized as a multidimensional construct that reflects consumers’ overall evaluation based on three key aspects: (1) aesthetic value (e.g., design, style, attractiveness), (2) functional value (e.g., comfort, quality, usability), and (3) religious value (e.g., compliance with Islamic principles of modesty). This integrated perspective is particularly relevant in the modest fashion context, where consumption decisions are influenced by both symbolic and utilitarian considerations.

Prior studies in halal-related products, such as cosmetics and food, have shown that positive attitudes significantly influence consumers’ decisions ( Ashraf, 2019; Nguyen et al., 2024; Sholihah et al., 2025; Sumi & Ahmed, 2022). A favorable attitude toward modest fashion, therefore, is expected to increase the likelihood of making purchase decisions.

H1:
*Attitude has a positive effect on purchase decision in modest fashion.*

### 2.2 Electronic Word of Mouth (E-WOM) and purchase decision

E-WOM refers to consumer-to-consumer communication conducted through digital platforms, where individuals share opinions, reviews, and recommendations about products and services ( Shen, 2021). It represents a modern form of interpersonal communication where individuals actively share product reviews, recommendations, and personal experiences across various online channels such as social media, discussion forums, blogs, and e-commerce platforms ( Akdim, 2021).

In contemporary consumer behavior, E-WOM has become a powerful source of influence that often shapes perceptions, attitudes, and purchase decisions. Consumers today tend to rely heavily on the experiences and evaluations of others before deciding to buy a product. Positive E-WOM can enhance trust, build credibility, and strengthen brand reputation, while negative E-WOM may create doubt, damage brand image, and discourage potential buyers ( Thurau et al., 2004; Yang, 2022).

E-WOM functions as a form of social proof that reduces uncertainty in decision-making. When consumers are exposed to consistent positive messages about a product, they are more likely to perceive it as reliable and worthy of purchase. Conversely, repeated exposure to negative comments may lead to avoidance behavior, regardless of the product’s actual quality ( Gaffar & Sentika, 2024; Sousa & Fortes, 2023). In the context of modest fashion, E-WOM plays a significant role in influencing how Muslim consumers evaluate and select clothing brands that align with their values. Through online discussions, consumers share insights not only about design and quality but also about sharia compliance, modesty, and ethical production. This exchange of information builds a sense of trust and community among consumers who share similar religious and moral orientations.

In the modest fashion industry, E-WOM plays a crucial role in reducing uncertainty and helping consumers evaluate product quality, brand reputation, and compatibility with their personal and religious values. Empirical studies ( Dehyadegari et al., 2025; Matiukaite et al., 2024; Singh et al., 2023; Yang, 2022) consistently show that E-WOM has a positive effect on purchase intention and decision, particularly for products that involve religious and symbolic attributes.

H2:
*E-WOM has a positive effect on purchase decision in modest fashion.*

### 2.3 Religiosity as a moderator

Religiosity can be viewed as the extent to which an individual lives out the values, beliefs, and practices rooted in their faith ( Safrilsyah et al., 2021). It represents not only one’s acknowledgment of religious teachings but also the depth of internalization that shapes attitudes, perceptions, and daily conduct ( Samad et al., 2022). In essence, religiosity reflects the degree to which religion becomes a central and guiding force in one’s life, affecting how a person interprets right and wrong, makes moral judgments, and interacts with the surrounding environment ( Abdullah et al., 2021).

From an Islamic perspective, religiosity is reflected through the observance of sharia principles in all aspects of life, including consumption ( Aruan & Wirdania, 2020). This means that the act of purchasing is not merely an economic transaction but also an expression of obedience to Allah. For Muslim consumers, the selection of goods and services must align with Islamic teachings that emphasize halal, tayyib (good and wholesome), and modest conduct. As a result, religiosity becomes a central determinant of consumer behavior, shaping preferences toward products that embody spiritual meaning and religious compliance ( Alsaad et al., 2021; Dehyadegari et al., 2025). When consumers are highly religious, their purchase decisions are likely to be filtered through Islamic principles, such as modesty, syariah compliance, and moral appropriateness. Consumers with a positive attitude toward modest fashion may be more likely to translate that attitude into an actual decision if their religiosity reinforces the belief that modest fashion aligns with Islamic teachings.

Findings from previous studies indicate that religiosity can strengthen the influence of independent variables on purchase decisions, particularly attitude and electronic word of mouth (E-WOM) ( Akhtar et al., 2020; Dehyadegari et al., 2025; Nickerson et al., 2023). Consumers with a high level of religiosity tend to filter their evaluations and decisions through religious principles, ensuring that their choices align with faith-based values. When exposed to positive attitudes or favorable online recommendations that reflect Islamic values, these consumers are more likely to translate such influences into actual purchasing behavior. Thus, religiosity not only shapes the moral foundation of consumer behavior but also reinforces the impact of both attitude and E-WOM on purchase decisions, leading to more consistent and value-driven consumption patterns.

H3:
*Religiosity moderates the relationship between attitude and purchase decision in modest fashion.*

H4:
*Religiosity moderates the relationship between E-WOM and purchase decision in modest fashion.*

**Figure 1.**
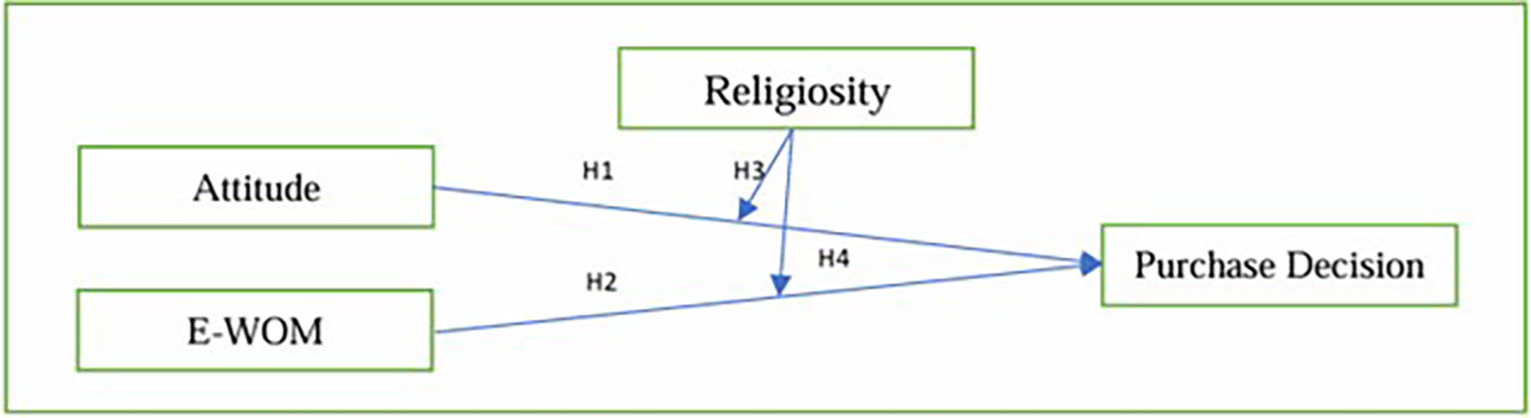
Conceptual model of research. Source: Author’s own work, 2025.

The conceptual model developed in this study is presented in Figure 1. It outlines the proposed relationships between Attitude, E-WOM, Religiosity, and Purchase Decision, and serves as the basis for formulating the research hypotheses.

## 3. Research methodology

### 3.1 Research design

This study employs a quantitative research design using Structural Equation Modeling (SEM) to examine how religiosity moderates the influence of attitude and electronic word of mouth (E-WOM) on modest fashion purchase decisions among Muslim consumers in Indonesia. The SEM approach was chosen because it enables simultaneous testing of multiple relationships among latent variables, allowing for a comprehensive understanding of the structural and measurement models within a single framework.

### 3.2 Population and sample

The population of this research consists of Muslim consumers residing in Jakarta and its surrounding areas who have experience in purchasing modest fashion products. Jakarta and its surrounding areas were selected as the research setting due to their role as the primary hub of Indonesia’s fashion industry and digital economy. The region represents a highly dynamic market where consumers are more exposed to modest fashion trends, social media influence, and diverse brand offerings. Therefore, it provides a relevant context for examining the interaction between attitude, E-WOM, and religiosity.

Respondents were selected using a non-probability sampling technique, specifically convenience sampling, as the questionnaire was distributed online via social media platforms. While this approach enables efficient data collection, it may limit the generalizability of the findings.

The determination of the sample size followed, which recommends a sample size of five to ten times the number of indicators used in the model. This study employed 36 indicators; hence, the minimum required sample ranged from 180 to 360 respondents. Data were collected through an online questionnaire distributed via social media channels. A total of 324 responses were received, but four were excluded due to incomplete answers, leaving 320 valid responses for analysis. This sample size meets the SEM requirements and ensures adequate statistical power.

This study did not involve medical procedures, clinical interventions, or the collection of sensitive personal data. Therefore, formal ethical approval from an institutional review board was not required according to the applicable national research ethics guidelines for social science research. Nevertheless, the study was conducted in full compliance with the ethical principles outlined in the Declaration of Helsinki. Participation was voluntary and anonymous. Informed consent was obtained through a digital consent mechanism embedded in the online questionnaire (Google Forms). At the beginning of the survey, respondents were presented with an online consent statement outlining the purpose of the study, confidentiality of responses, and their right to withdraw at any time without consequences. Respondents were required to actively indicate their agreement by selecting “Yes” before accessing the research questions. Those who selected “No” were automatically directed to exit the questionnaire and could not proceed further.

### 3.3 Measurement and instrument

All constructs were measured using multi-item scales adapted from prior validated studies. Attitude was measured using items adapted from Ajzen (1991) and Sumarliah et al. (2022), reflecting cognitive, affective, and conative dimensions. The attitude construct was operationalized to capture aesthetic, functional, and religious evaluations of modest fashion products, ensuring alignment with the multidimensional nature of consumer perception in Islamic fashion contexts. E-WOM was measured based on Akdim (2021) and Thurau et al. (2004), capturing message credibility, quality, and recommendation behavior. Religiosity was operationalized using scales adapted from Aruan and Wirdania (2020) and Abdullah et al. (2021), covering belief, practice, and ethical dimensions. Purchase decision was measured using indicators adapted from Ashraf (2019) and Nguyen et al. (2024), reflecting both intention and actual purchase behavior. The purchase decision construct reflects actual or realized consumer behavior rather than mere intention, capturing the extent to which consumers have made or are committed to making a purchase.

### 3.4 Data analysis

Data were analyzed using Partial Least Squares Structural Equation Modeling (PLS-SEM) with SmartPLS version 4. This technique was selected due to its suitability for exploratory and predictive research involving complex models and moderating effects. The analysis proceeded in two main stages: the measurement model assessment and the structural model assessment.

The measurement model was evaluated through indicator reliability, internal consistency (Cronbach’s alpha and composite reliability), convergent validity (average variance extracted or AVE), and discriminant validity. The structural model was then examined using path coefficients, coefficient of determination (R ^2^), effect size (f ^2^), and predictive relevance (Q ^2^). The moderating role of religiosity was tested using an interaction term between religiosity and the predictor variables (attitude and E-WOM), and its significance was evaluated using the bootstrapping method with 5,000 resamples. To assess the potential for Common Method Bias (CMB), this study employed the full collinearity variance inflation factor (VIF) approach as recommended for PLS-SEM. A VIF value below 3.3 indicates that the model is free from common method bias. In addition to the full collinearity VIF assessment, Harman’s single-factor test was conducted to further examine the presence of common method bias.

## 4. Research finding

### 4.1 Measurement model

The measurement model was assessed to ensure the reliability and validity of all constructs before proceeding to structural model testing. Table 1 presents the results of the measurement model evaluation, which includes factor loadings, Cronbach’s alpha, composite reliability, and average variance extracted (AVE) for each construct.

**Table 1.** Measurement model result.

Constructs	Item	Loading	Cronbach alpha	Composite reliability	AVE
Purchase Decision	Y1	0.906	0.974	0.975	0.778
	Y2	0.880			
	Y3	0.897			
	Y4	0.908			
	Y5	0.924			
	Y6	0.814			
	Y7	0.852			
	Y8	0.889			
	Y9	0.870			
	Y10	0.892			
	Y11	0.894			
	Y12	0.851			
Attitude	X1.1	0.890	0.951	0.952	0.804
	X1.2	0.859			
	X1.3	0.900			
	X1.4	0.889			
	X1.5	0.931			
	X1.6	0.911			
E-WOM	X2.1	0.940	0.961	0.963	0.788
	X2.2	0.846			
	X2.3	0.915			
	X2.4	0.838			
	X2.5	0.902			
	X2.6	0.868			
	X2.7	0.884			
	X2.8	0.905			
Religiosity	M1.1	0.889	0.977	0.977	0.826
	M1.2	0.902			
	M1.3	0.918			
	M1.4	0.905			
	M1.5	0.895			
	M1.6	0.930			
	M1.7	0.900			
	M1.8	0.884			
	M1.9	0.906			
	M1.10	0.958			

Source: Author’s own work based on the analysis of collected data, 2025.

All item loadings exceeded the minimum threshold value of 0.70, indicating that each indicator had a strong relationship with its respective latent construct. The purchase decision construct showed item loadings ranging from 0.814 to 0.924, suggesting that all indicators consistently represented consumers’ purchasing behavior toward modest fashion products. Similarly, the attitude construct exhibited loadings between 0.859 and 0.931, demonstrating that the items effectively captured respondents’ cognitive and affective evaluations. The E-WOM construct recorded loadings between 0.838 and 0.940, reflecting strong consistency in measuring message quality, credibility, and recommendation behavior. Finally, the religiosity construct displayed loadings ranging from 0.884 to 0.958, confirming that all items were highly representative of the dimensions of Islamic belief, worship, and ethical conduct.

Internal consistency reliability was confirmed through Cronbach’s alpha and composite reliability (CR) values, all of which exceeded the acceptable threshold of 0.70. Specifically, Cronbach’s alpha values ranged from 0.951 (attitude) to 0.977 (religiosity), while CR values varied between 0.952 and 0.977. These results indicate that the measurement model demonstrated a high level of internal consistency across all constructs.

Convergent validity was evaluated using the Average Variance Extracted (AVE). All constructs showed AVE values above the recommended minimum of 0.50, confirming that each construct explains more than half of the variance of its indicators. The AVE values were 0.778 for purchase decision, 0.804 for attitude, 0.788 for E-WOM, and 0.826 for religiosity, signifying that the constructs possess satisfactory convergent validity.

### 4.2 Structural model

To assess the potential for Common Method Bias (CMB), this study employed the full collinearity variance inflation factor (VIF) approach and Harman’s single-factor test. The full collinearity assessment showed that the VIF values ranged from 24.759 to 33.237, indicating high collinearity among several constructs. In addition, Harman’s single-factor test revealed that the first factor accounted for 78.383% of the total variance. These findings suggest the presence of potential common method bias, which may be associated with the use of self-reported cross-sectional data and highly homogeneous respondent perceptions. Nevertheless, the structural relationships remained theoretically meaningful and statistically significant. Therefore, the findings should be interpreted with caution, and future studies are encouraged to employ longitudinal or multi-source data collection designs to minimize potential method bias.

After confirming the validity and reliability of the measurement model, the structural model was analyzed to examine the relationships among latent constructs and to evaluate the predictive strength of the model. The analysis included the assessment of the coefficient of determination (R ^2^), effect size (f ^2^), and the significance of the path coefficients.

**Figure 2.**
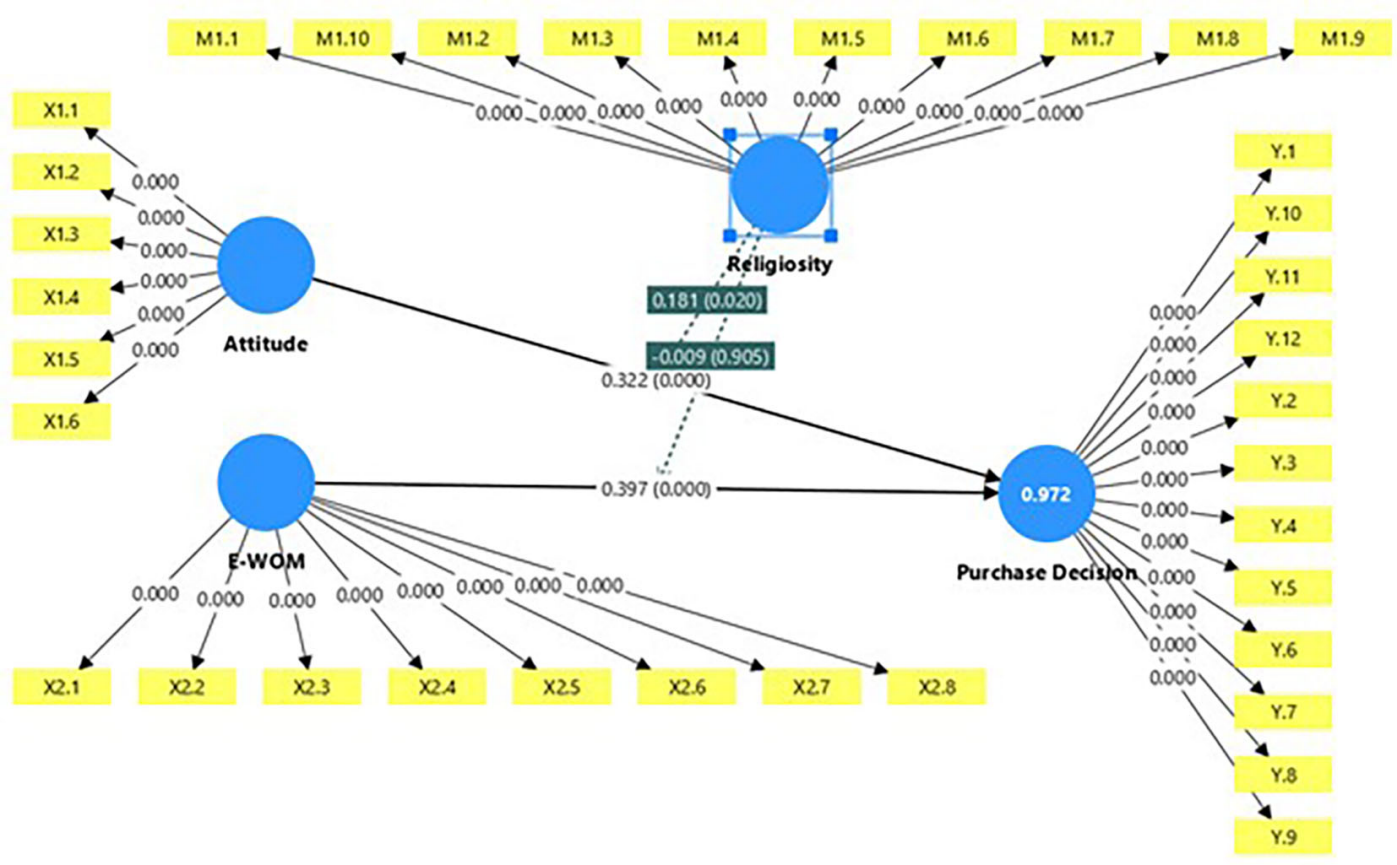
Hypothesis testing results. Source: Author’s own work based on the analysis of collected data, 2025.

As illustrated in Figure 2, the path from Attitude to Purchase Decision shows a positive but statistically insignificant effect, while E-WOM demonstrates a significant influence. The figure also indicates the mediating role of Religiosity in the model.

The R ^2^ value for the purchase decision construct was 0.972, with an adjusted R ^2^ of 0.971, summarized in Table 2. This result indicates that attitude, E-WOM, and religiosity, including their interaction terms, jointly explain 97.2% of the variance in modest fashion purchase decisions. The predictive relevance of the model was assessed using the Stone-Geisser Q ^2^ value. The analysis showed that the Q ^2^ value for Purchase Decision was 0.964, indicating strong predictive relevance because the value exceeded zero. Furthermore, the PLSpredict assessment demonstrated adequate predictive performance, as the prediction error generated by the PLS model remained relatively low (MSE = 0.0079), indicating satisfactory out-of-sample predictive capability.

**Table 2.** Common method bias, predictive relevance, and model fit evaluation.

Evaluation criteria	Result	Threshold	Interpretation
Harman’s Single Factor	78.383%	<50%	Potential common method bias detected
Full Collinearity VIF	24.759–33.237	<3.3	High collinearity among constructs
R ^2^ Purchase Decision	0.972	>0.67	Substantial explanatory power
Adjusted R ^2^	0.971	>0.67	Strong model consistency
Q ^2^ Purchase Decision	0.964	>0	Strong predictive relevance
PLSpredict MSE	0.0079	Lower is better	Adequate predictive performance
SRMR	<0.08	<0.08	Acceptable model fit

Source: Author’s own work based on the analysis of collected data, 2025.

The effect size (f ^2^) values show the contribution of each predictor to the endogenous construct. E-WOM recorded the highest effect (f ^2^ = 0.203), indicating that electronic word of mouth exerts the strongest influence on purchase decisions. Attitude also showed a meaningful contribution (f ^2^ = 0.105), suggesting that consumers’ positive evaluations toward modest fashion significantly enhance their purchasing decisions. Religiosity demonstrated a smaller but relevant effect (f ^2^ = 0.062), confirming its role as an important personal determinant of purchasing behavior. The interaction between religiosity and attitude yielded a small but notable effect (f ^2^ = 0.044), while the interaction between religiosity and E-WOM had a negligible effect (f ^2^ = 0.000), indicating that religiosity only moderates the influence of attitude, not E-WOM.

The path coefficient results, summarized in Table 3, further confirm these findings. Attitude toward modest fashion had a significant positive effect on purchase decision (β = 0.322, t = 3.870, p < 0.001). Likewise, E-WOM showed a strong and significant positive relationship with purchase decision (β = 0.397, t = 5.012, p < 0.001). Religiosity also exerted a significant direct influence on purchase decision (β = 0.231, t = 2.731, p = 0.006), highlighting that higher religiosity levels are associated with stronger purchasing decisions toward modest fashion.

**Table 3.** Structural model result.

Constructs	f square	t statistics	p value
Attitude -> Purchase Decision	0.105	3.870	0.000
E-WOM -> Purchase Decision	0.203	5.012	0.000
Religiosity -> Purchase Decision	0.062	2.731	0.006
Religiosity x Attitude -> Purchase Decision	0.044	2.322	0.020
Religiosity x E-WOM -> Purchase Decision	0.000	0.119	0.905

Source: Author’s own work based on the analysis of collected data, 2025.

The moderating analysis revealed that religiosity significantly strengthened the effect of attitude on purchase decision (β = 0.181, t = 2.322, p = 0.020), indicating that the more religious the consumers are, the more consistent their positive attitudes become in translating into actual purchasing behavior. However, the interaction between religiosity and E-WOM was statistically insignificant (β = -0.009, t = 0.119, p = 0.905), suggesting that religiosity does not moderate the relationship between E-WOM and purchase decision.

These findings confirm that attitude, E-WOM, and religiosity play significant roles in shaping modest fashion purchase decisions among Muslim consumers in Indonesia. Attitude and E-WOM remain the most dominant predictors, while religiosity acts as a reinforcing factor that enhances the effect of attitude but not E-WOM.

The predictive relevance of the model was assessed using the Stone-Geisser Q ^2^ value. The results show that the Q ^2^ value for purchase decision is greater than zero, indicating that the model has strong predictive relevance. Additionally, PLSpredict analysis was conducted to evaluate the model’s out-of-sample predictive power. The results demonstrate that the model achieves adequate predictive performance, as the prediction errors are lower than those of the benchmark linear model. Furthermore, model fit was assessed using the Standardized Root Mean Square Residual (SRMR). The SRMR value was below the recommended threshold of 0.08, indicating a good model fit.

## 5. Discussion

The results of this study provide meaningful insights into how religiosity shapes consumers’ purchase decisions in the modest fashion market in Indonesia. Attitude and electronic word of mouth (E-WOM) emerged as significant predictors of purchase intention, reaffirming established consumer behavior theory that positive attitudes and peer recommendations strongly influence purchase decisions. This finding is consistent with prior studies indicating that consumer attitudes toward modest fashion brands are often built upon both functional and symbolic values that align with Islamic principles ( Hwang & Kim, 2020, 2021; Mirza, 2024). When consumers perceive that a product not only satisfies their aesthetic or quality expectations but also reflects their religious commitment, their attitude becomes a key driver in forming purchase intentions. Importantly, religiosity not only directly affects decision-making but also strengthens the relationship between attitude and purchase intention. This suggests that consumers with deep religious values weigh their positive attitudes towards modest fashion more heavily when making purchase decisions, reflecting an alignment of personal beliefs and consumption behaviors.

In the context of Indonesia’s modest fashion market, religiosity is not just an additional factor but a fundamental basis for consumers when making purchase decisions ( Kusumawati et al., 2019; Munawaroh et al., 2025). Consumers who firmly hold religious values tend to give greater weight to their positive attitudes toward products when deciding what to buy. This means that purchases are influenced not only by trends or style but also by alignment with their beliefs and religious identity.

In practice, religiosity encourages consumers to be more selective and discerning, choosing fashion items that not only meet aesthetic standards but also comply with syariah principles, such as modesty and the halal status of materials or manufacturing processes. This trust arising from alignment is reinforced by recommendations from close peers, both directly and through social media (E-WOM), which plays a significant role in shaping purchase intention. However, it is important to recognize that younger consumers increasingly balance religious values with lifestyle and fashion trends. This requires businesses to offer products that fulfill religious demands while also being attractive in terms of style and relevant to contemporary tastes.

On the other hand, religiosity did not moderate the effect of E-WOM on purchase decisions. This indicates that regardless of religious intensity, electronic word of mouth operates independently as a powerful driver. This means that no matter how deeply religious someone is, the influence of online reviews, recommendations, and social sharing remains strong and independent. The power of social proof and peer opinions in the digital world cuts across all levels of religious commitment. It highlights the universal influence of social proof and online peer opinions in the digital era, impacting consumers across the religiosity spectrum ( Bakar et al., 2013; Misiak & Paruzel-Czachura, 2025). This finding aligns with ( Millie & Baulch, 2024) work on digital influence in Islamic consumerism, which argues that the increasing visibility of pious consumption and the uptake of new technologies by Indonesian Muslims illustrate how the borders between religion and consumerism have become increasingly porous. This suggests that religious and non-religious cultural forms are blending, which can facilitate the transcendence of religious differences in consumer behavior.

## 6. Conclusion and suggestion

This study provides empirical insights into how attitude, electronic word of mouth (E-WOM), and religiosity shape Muslim consumers’ purchasing behavior in Indonesia’s modest fashion market. The results confirm that both attitude and E-WOM have significant and positive effects on purchase decisions, demonstrating that cognitive and social influences remain central in explaining consumer behavior within Islamic markets. Among the predictors, E-WOM emerged as the strongest determinant, underscoring the growing role of digital peer communication in driving consumer confidence and reducing uncertainty in the decision-making process. Religiosity also exhibits a direct positive influence on purchase decision, signifying that faith-based values remain a vital part of consumption patterns among Muslim consumers. Moreover, religiosity strengthens the link between attitude and purchase decision, implying that higher religious commitment enhances the translation of favorable attitudes into actual purchasing behavior. However, religiosity does not moderate the relationship between E-WOM and purchase decision, indicating that digital influence operates across varying levels of religious commitment and reflects a broader social mechanism in the digital era.

Theoretically, this study contributes to Islamic marketing literature by extending the application of the Theory of Planned Behavior (TPB) within the context of modest fashion consumption. It demonstrates that religiosity not only serves as a direct determinant but also as a moderating variable that reinforces attitude-driven decision-making among Muslim consumers. These findings offer a nuanced understanding of how religious identity interacts with psychological and technological factors in shaping market behavior in the halal economy.

From a managerial perspective, the findings highlight the importance for modest fashion entrepreneurs and marketers to integrate religiosity and digital engagement in their strategies. Marketers should emphasize syariah compliance, ethical sourcing, and value-based storytelling to strengthen consumer attitudes rooted in Islamic principles. At the same time, they must cultivate credible E-WOM by encouraging authentic consumer reviews, influencer collaborations, and social media interactions that foster trust and community. Balancing spiritual authenticity with contemporary design and digital appeal will allow brands to effectively connect with both religiously driven and trend-oriented consumers. For policymakers, the findings underscore the importance of supporting the modest fashion industry as part of Indonesia’s broader halal economy agenda, by promoting digital literacy, ethical standards, and market competitiveness.

## 7. Limitation and future research

This study is subject to potential common method bias due to the use of self-reported data collected from a single source. Although statistical remedies were applied, future research is encouraged to use longitudinal or multi-source data to minimize this issue. This study is subject to limitations related to the use of convenience sampling, which may introduce selection bias and limit the generalizability of the findings. Although the reliability values are very high, exceeding 0.95, this may indicate potential item redundancy. Future studies are encouraged to refine the measurement instrument by reducing overlapping items to enhance content validity.

However, the focus on Jakarta limits the generalizability of the findings, as consumer behavior in other regions of Indonesia may differ due to variations in cultural norms, income levels, and degrees of religiosity. Future studies are encouraged to include a more diverse geographical sample.

Future research also could extend this study by examining generational or cross-cultural variations in the interplay between religiosity and digital influence. Comparative studies between Muslim-majority and minority contexts would enrich understanding of how Islamic values, social identity, and technological engagement shape consumer behavior across different cultural settings.

## Data Availability

Zenodo. *Quesioner and Data of Research.* https://doi.org/10.5281/zenodo.17958807 ( Fathoni, M. A. 2025). This project contains the following underlying data:
• Data of Research.csv: Raw survey data used in the analysis. • Quesioner.docx: Questionnaire instrument used for data collection. Data are available under the terms of the Creative Commons Attribution 4.0 International (CC-BY 4.0) license.
