# Supplementary material for: What Drives Indonesian People to Purchase Modest Fashion? The Moderating Role of Religiosity
Source: F1000Res. 2026 Sep 15;15:127. Originally published 2026 Jan 28. [Version 4] doi: 10.12688/f1000research.173033.4 (PMC13332377; doi:10.12688/f1000research.173033.4)
Supplement: Peer review history

# Reviewer report: Reviewer response for version 4

**DOI:** 10.5256/f1000research.209948.r521483

**Published:** 2026-09-28

**Authors:** Tiara Nur Anisah

**Affiliations:** 1 Janabadra University, Yogyakarta, Indonesia

I would like to thank the authors for their diligence and responsiveness in addressing the concerns raised in my previous review. I have reviewed Version 4 of the manuscript and noted the following improvements:

1. **Theoretical Framework:** The integration of the Extended Theory of Planned Behavior (TPB) is now explicitly articulated in the introduction. This provides a much stronger and clearer theoretical foundation for why E-WOM and Religiosity were added to the model.
2. **Methodological Transparency (HTMT):** I commend the authors for their academic transparency in calculating and reporting the Heterotrait-Monotrait Ratio (HTMT) values. While the values indicate substantial empirical overlap among the constructs (ranging from 0.999 to 1.023), retaining them based on theoretical distinctiveness while explicitly acknowledging this empirical limitation in Section 7 is the correct and ethical approach in quantitative reporting.
3. **Research Implications:** The conclusion section has been vastly improved. Separating the theoretical contributions from the managerial and policy implications provides clear, structured, and actionable takeaways for the readers.

The authors have successfully addressed all my previous reservations. The manuscript now meets the rigorous reporting standards of PLS-SEM and contributes meaningfully to the literature on Islamic marketing and consumer behavior. I have no further comments to make.

Is the work clearly and accurately presented and does it cite the current literature?

Partly

If applicable, is the statistical analysis and its interpretation appropriate?

Partly

Are all the source data underlying the results available to ensure full reproducibility?

Partly

Is the study design appropriate and is the work technically sound?

Partly

Are the conclusions drawn adequately supported by the results?

Partly

Are sufficient details of methods and analysis provided to allow replication by others?

No

Reviewer Expertise:

Marketing Management, Consumer Behavior, Marketing and Sustainability, Quantitative Methods (PLS-SEM)

I confirm that I have read this submission and believe that I have an appropriate level of expertise to confirm that it is of an acceptable scientific standard.

# Reviewer report: Reviewer response for version 3

**DOI:** 10.5256/f1000research.202552.r493562

**Published:** 2026-07-03

**Authors:** Izian Idris

**Affiliations:** 1 Taylor's Business School, Taylor's University, Subang Jaya, Indonesia

- **Title**: The title is descriptive but generic. It signals the topic and moderating variable but does not emphasize novelty or theoretical contribution. Consider sharpening it to highlight the unique angle (e.g., cultural, consumer psychology, or religiosity dimensions).
- **Abstract**: The abstract provides a basic overview but fails to articulate the originality of the study. It should clearly state the research gap, contribution, and implications rather than just summarizing the process.
- **Research Background**: The background is relevant but too descriptive. It reiterates known facts about modest fashion and religiosity without identifying a specific gap in Indonesian consumer behavior studies.
- **Problem Statement**: Although a problem statement exists, it is vague and lacks precision. It should explicitly explain *why* this study is necessary, what gap it fills, and how religiosity as a moderator adds new insight. Right now, the justification for originality is weak.
- **Literature Review**: The review is not critical enough. It summarizes prior studies but does not analyze contradictions, underexplored areas, or theoretical tensions.
- Improvement: Focus on critically evaluating existing studies, highlighting gaps in modest fashion research in Indonesia, and showing how religiosity has been underexamined in consumer behavior.
- Even with limited prior studies, the authors should leverage this scarcity to emphasize the novelty of their contribution.
- **Methodology**: The methodology is acceptable but lacks clarity. The rationale for choosing specific methods is not well explained. Readers should understand *why* these methods are appropriate for testing the moderating role of religiosity.
- **Analysis**: The analysis is presented but superficial. Statistical techniques are not justified, and interpretation is limited. More robust explanation of the analytical approach and its alignment with research objectives is needed.
- **Discussion**: The discussion is too surface-level. It restates findings without connecting them to broader theoretical debates or practical implications.
- **Conclusion**: The conclusion is generic and does not highlight the study’s contribution. It should emphasize how the findings advance knowledge on religiosity and consumer behavior in modest fashion.
- **Implications**: No implications are stated. This is a major weakness. The paper should provide:
- **Theoretical implications**: How does this study extend consumer behavior or religiosity theory?
- **Practical implications**: What can fashion brands, marketers, or policymakers learn from this?
- **Societal implications**: How does this research inform cultural or religious perspectives in Indonesia?

Is the work clearly and accurately presented and does it cite the current literature?

Partly

If applicable, is the statistical analysis and its interpretation appropriate?

Yes

Are all the source data underlying the results available to ensure full reproducibility?

Partly

Is the study design appropriate and is the work technically sound?

Partly

Are the conclusions drawn adequately supported by the results?

Partly

Are sufficient details of methods and analysis provided to allow replication by others?

No

Reviewer Expertise:

Marketing

I confirm that I have read this submission and believe that I have an appropriate level of expertise to confirm that it is of an acceptable scientific standard, however I have significant reservations, as outlined above.

# Response

**Published:** 2026-09-04

**Authors:** Muhammad Anwar Fathoni

**Affiliations:** Islamic Economics, Universitas Pembangunan Nasional Veteran Jakarta, Jakarta, Special Capital Region of Jakarta, Indonesia

Thank you for the constructive comments. We have revised the manuscript to strengthen its originality, research gap, theoretical positioning, methodological justification, and discussion of the findings. The title and abstract have been sharpened, while the background and literature review now more clearly highlight the research gap concerning religiosity and modest fashion consumer behaviour in Indonesia. We have also expanded the theoretical, practical, and societal implications and strengthened the conclusion accordingly. These revisions focus on the framing, interpretation, and presentation of the study, while the data, research model, and empirical results remain unchanged.

# Reviewer report: Reviewer response for version 3

**DOI:** 10.5256/f1000research.202552.r492511

**Published:** 2026-06-27

**Authors:** Tiara Nur Anisah

**Affiliations:** 1 Janabadra University, Yogyakarta, Indonesia

Thank you to the authors for the significant improvements made in this third version, particularly regarding the transparent reporting of the Common Method Bias (CMB) metrics and predictive relevance. However, evaluating the manuscript against the rigorous standards of PLS-SEM reporting and high-impact journal structures reveals three critical omissions that must be addressed before this manuscript can be fully approved:

**1. Absence of a Dedicated "Grand Theory" Section**

 While the Theory of Planned Behavior (TPB) is briefly mentioned in the Introduction, the Literature Review (Section 2) abruptly jumps into variable relationships (Attitude, E-WOM, Religiosity) without a cohesive theoretical foundation. The authors must add a dedicated sub-section at the beginning of the Literature Review (e.g., *2.1. The Extended Theory of Planned Behavior*) to explicitly explain the grand theory. This section must theoretically justify how and why E-WOM and Religiosity are integrated into the traditional TPB framework in this specific context.

**2. Missing Discriminant Validity (HTMT) in the Measurement Model**

 In Section 4.1, the authors successfully reported internal consistency (Cronbach's Alpha, CR) and convergent validity (AVE). However, there is an absolute absence of Discriminant Validity reporting. In modern PLS-SEM standards, establishing discriminant validity is mandatory before proceeding to the structural model. The authors must calculate and report the Heterotrait-Monotrait Ratio (HTMT) values to prove that the constructs are empirically distinct.

**3. Underdeveloped Research Implications**

 In Section 6, the theoretical and managerial implications are merged into a single, brief paragraph at the end of the conclusion. For a paper of this scope, the implications need to be substantially expanded and structured. Please separate the Theoretical Implications and Managerial Implications into distinct paragraphs or sub-sections and elaborate more deeply on how fashion marketers or policymakers can practically apply these findings.

 Addressing these three specific points will significantly elevate the academic rigor of the manuscript and satisfy the standard reporting guidelines for PLS-SEM. I look forward to reviewing the final revisions.

Is the work clearly and accurately presented and does it cite the current literature?

Partly

If applicable, is the statistical analysis and its interpretation appropriate?

Partly

Are all the source data underlying the results available to ensure full reproducibility?

Partly

Is the study design appropriate and is the work technically sound?

Partly

Are the conclusions drawn adequately supported by the results?

Partly

Are sufficient details of methods and analysis provided to allow replication by others?

No

Reviewer Expertise:

Marketing Management, Consumer Behavior, Marketing and Sustainability, Quantitative Methods (PLS-SEM)

I confirm that I have read this submission and believe that I have an appropriate level of expertise to confirm that it is of an acceptable scientific standard, however I have significant reservations, as outlined above.

# Response

**Published:** 2026-09-04

**Authors:** Muhammad Anwar Fathoni

**Affiliations:** Islamic Economics, Universitas Pembangunan Nasional Veteran Jakarta, Jakarta, Special Capital Region of Jakarta, Indonesia

Dear Reviewer

 Thank you for this important suggestion. We have now added the HTMT assessment and HTMT inference results to the revised manuscript. The results show relatively high HTMT values among Attitude, E-WOM, Purchase Decision, and Religiosity, indicating substantial empirical associations among these constructs. Rather than modifying the measurement model solely to reduce the HTMT values, we retain the theoretically specified constructs and explicitly discuss the observed empirical overlap as a limitation of the study. The corresponding results and discussion have been added to the revised manuscript.

# Reviewer report: Reviewer response for version 2

**DOI:** 10.5256/f1000research.200523.r483948

**Published:** 2026-05-25

**Authors:** A'rasy fahrullah

**Affiliations:** 1 Universitas Negeri Surabaya, Surabaya, East Java, Indonesia

no further comments

Is the work clearly and accurately presented and does it cite the current literature?

Yes

If applicable, is the statistical analysis and its interpretation appropriate?

I cannot comment. A qualified statistician is required.

Are all the source data underlying the results available to ensure full reproducibility?

No

Is the study design appropriate and is the work technically sound?

Yes

Are the conclusions drawn adequately supported by the results?

Yes

Are sufficient details of methods and analysis provided to allow replication by others?

Yes

Reviewer Expertise:

islamic economics

I confirm that I have read this submission and believe that I have an appropriate level of expertise to confirm that it is of an acceptable scientific standard.

# Reviewer report: Reviewer response for version 2

**DOI:** 10.5256/f1000research.200523.r483949

**Published:** 2026-05-25

**Authors:** Tiara Nur Anisah

**Affiliations:** 1 Janabadra University, Yogyakarta, Indonesia

Thank you for submitting the revised version of your manuscript. I appreciate the effort you have put into addressing my previous concerns. The theoretical integration is now clearer, the measurement instruments are properly cited, the sampling methodology has been accurately corrected, and I commend your transparency in acknowledging the potential item redundancy in the limitations section.

 However, while you have addressed the methodological and statistical points in the text, **the reporting of the PLS-SEM structural model evaluation remains incomplete because the actual statistical values and tables are missing.** To achieve full approval, please address the following specific reporting issues:

**1. Missing Statistical Values for CMB Testing:** In Section 4.2, you stated that some VIF values exceeded the threshold and that Harman's single-factor test accounted for "less than 50%" of the variance. In rigorous PLS-SEM reporting, you must explicitly state the actual values. What were the specific VIF values? What was the exact percentage of variance extracted in Harman's test (e.g., 42.5%)?

**2. Missing Values for Predictive Relevance and Model Fit:** You narrated that the Q² value is greater than zero, the PLSpredict errors are lower than the benchmark, and the SRMR is below 0.08. Academic rigor requires you to report the precise numerical values for these metrics. Please include these specific figures in the text (e.g., SRMR = 0.054, exact Q² values) or add a brief summary table for the structural model evaluation.

 Once these specific numerical values are explicitly reported to support your narrative claims, I will be more than happy to fully approve the manuscript.

Is the work clearly and accurately presented and does it cite the current literature?

Partly

If applicable, is the statistical analysis and its interpretation appropriate?

Partly

Are all the source data underlying the results available to ensure full reproducibility?

Partly

Is the study design appropriate and is the work technically sound?

Partly

Are the conclusions drawn adequately supported by the results?

Partly

Are sufficient details of methods and analysis provided to allow replication by others?

No

Reviewer Expertise:

Marketing Management, Consumer Behavior, Marketing and Sustainability, Quantitative Methods (PLS-SEM)

I confirm that I have read this submission and believe that I have an appropriate level of expertise to confirm that it is of an acceptable scientific standard, however I have significant reservations, as outlined above.

# Response

**Published:** 2026-05-26

**Authors:** Muhammad Anwar Fathoni

**Affiliations:** Islamic Economics, Universitas Pembangunan Nasional Veteran Jakarta, Jakarta, Special Capital Region of Jakarta, Indonesia

Thank you very much for the reviewer’s valuable comments and suggestions. We really appreciate the reviewer’s attention to the completeness and transparency of the PLS-SEM reporting.

 In response to the comments, we have revised Section 4.2 by explicitly reporting the statistical values related to common method bias testing, predictive relevance, and model fit evaluation.

 Specifically, we now report the exact values of the full collinearity VIF and Harman’s single-factor test. The results show that Harman’s single-factor test explained 78.383% of the total variance, while the VIF values ranged from 24.759 to 33.237. We acknowledge that these findings indicate potential common method bias and high collinearity among constructs. Therefore, we added a more transparent explanation in the manuscript and strengthened the limitation section to clarify this issue.

 In addition, we completed the structural model evaluation by reporting the Q², PLSpredict, and SRMR results. The Q² value for Purchase Decision was reported as 0.964, indicating strong predictive relevance. We also added information regarding PLSpredict performance and clarified that the SRMR value was below the recommended threshold of 0.08, indicating acceptable model fit.

 To improve clarity and readability, we also added a new table entitled “Common Method Bias, Predictive Relevance, and Model Fit Evaluation” to summarize the key structural model assessment results more clearly.

 We sincerely thank the reviewer once again for the constructive feedback, which has helped us improve the rigor and transparency of the manuscript.

# Reviewer report: Reviewer response for version 1

**DOI:** 10.5256/f1000research.190807.r464457

**Published:** 2026-03-31

**Authors:** A'rasy fahrullah

**Affiliations:** 1 Universitas Negeri Surabaya, Surabaya, East Java, Indonesia

This manuscript addresses a relevant topic in Indonesia’s modest fashion market. However, several issues require clarification. First, although "modest fashion" is widely used, its conceptual boundary is not clearly defined in this study. Second, the operationalization of the "attitude" construct lacks transparency, particularly regarding whether it reflects religious, aesthetic, or functional evaluation. Third, the manuscript inconsistently uses "purchase decision", "intention", and "behavior", which should be clearly distinguished. Finally, the exclusive focus on Jakarta respondents is not sufficiently justified and may limit generalizability, given Indonesia’s diverse socio-cultural context. Addressing these points would improve conceptual clarity and robustness.

Is the work clearly and accurately presented and does it cite the current literature?

Yes

If applicable, is the statistical analysis and its interpretation appropriate?

I cannot comment. A qualified statistician is required.

Are all the source data underlying the results available to ensure full reproducibility?

No

Is the study design appropriate and is the work technically sound?

Yes

Are the conclusions drawn adequately supported by the results?

Yes

Are sufficient details of methods and analysis provided to allow replication by others?

Yes

Reviewer Expertise:

islamic economics

I confirm that I have read this submission and believe that I have an appropriate level of expertise to confirm that it is of an acceptable scientific standard, however I have significant reservations, as outlined above.

# Response

**Published:** 2026-04-28

**Authors:** Muhammad Anwar Fathoni

**Affiliations:** Islamic Economics, Universitas Pembangunan Nasional Veteran Jakarta, Jakarta, Special Capital Region of Jakarta, Indonesia

We thank the reviewer for highlighting important issues related to conceptual clarity. In response, we have made several improvements. First, we have clearly defined “modest fashion” and its conceptual boundaries in the Introduction. Second, we clarified the operationalization of the attitude construct by specifying its aesthetic, functional, and religious dimensions. Third, we ensured consistency by distinguishing purchase decision from purchase intention and behavior, and clearly positioning it as a behavioral outcome. Finally, we provided a stronger justification for focusing on Jakarta as the research setting and acknowledged its limitation in terms of generalizability. These revisions significantly enhance the conceptual rigor of the manuscript.

# Reviewer report: Reviewer response for version 1

**DOI:** 10.5256/f1000research.190807.r466472

**Published:** 2026-03-18

**Authors:** Tiara Nur Anisah

**Affiliations:** 1 Janabadra University, Yogyakarta, Indonesia

The study addresses an interesting and relevant topic within the growing modest fashion industry in Indonesia. The use of Partial Least Squares Structural Equation Modeling (PLS-SEM) to test the moderating role of religiosity adds empirical value. However, the manuscript requires substantial revisions before it can be fully approved. The conceptual model lacks a cohesive theoretical foundation, the measurement development is untraceable, and the PLS-SEM reporting fails to meet the current standards of rigor for high-impact journals (e.g., missing CMB testing and predictive relevance metrics).

**Major Revisions:**

 1. Restructuring the Introduction and Theoretical Framework:

- Theoretical Justification: The conceptual model (Figure 1) appears fragmented. The authors briefly mention the Theory of Planned Behavior (TPB), but they fail to theoretically justify *why* E-WOM and Religiosity are integrated into this specific model. The manuscript needs a robust theoretical argument explaining how these variables extend or interact within a unified framework.
- State of the Art & Novelty: The Introduction fails to establish a critical research gap. Stating that "limited attention has been given to modest fashion in Indonesia" is a contextual gap, not a theoretical one. The authors must critically debate previous literature, highlight theoretical conflicting findings or shortcomings, and explicitly state the conceptual novelty of this study.

**2. Measurement Instrument Traceability (Section 3.3):**

 The authors claim that "All constructs were measured using multi-item scales adapted from prior studies." However, no citations are provided to indicate which prior studies these scales were adapted from. For a Q1 journal, the operationalization of variables must be highly transparent. The authors must properly cite the original sources of their questionnaire items.

**3. Common Method Bias (CMB) Testing:**

 Given that the data is cross-sectional and collected via self-reported online surveys, Common Method Bias is a significant threat to the validity of the findings. The manuscript currently lacks any mention of CMB. The authors must perform and report a CMB test, such as the Full Collinearity VIF approach (often recommended in PLS-SEM) or Harman’s Single-Factor test, to prove that the data is free from this bias.

**4. Incomplete Structural Model Evaluation (PLS-SEM Rigor):**

 The reporting of the structural model in Section 4.2 is incomplete and falls short of modern PLS-SEM reporting guidelines (e.g., Hair et al., 2019/2022).

- Missing Q2 and PLSpredict: In Section 3.4, the authors state they will evaluate predictive relevance (Q2), but this is entirely missing from the Results section. The authors must report the Q2 predict values or utilize the PLSpredict procedure to demonstrate the model's out-of-sample predictive power.
- Model Fit: The authors should report the model fit metric, specifically the Standardized Root Mean Square Residual (SRMR), to demonstrate that the data fits the proposed model well.

**5. Contradiction in Sampling Methodology:**

 In Section 3.2, the authors claim to use a "random sampling technique." However, they subsequently state that data was collected via an "online questionnaire distributed via social media channels." Distributing a survey via social media without a defined sampling frame is *convenience* or *purposive sampling*, not probability/random sampling. The authors must correct this methodological claim and acknowledge its implications in the limitations section.

**6. Measurement Model (Item Redundancy):**

 In Table 1, the Cronbach's Alpha and Composite Reliability values are extraordinarily high (ranging from 0.951 to 0.977). While values above 0.70 are acceptable, values exceeding 0.95 strongly indicate item redundancy (the survey questions are likely asking the exact same thing with slight wording variations). The authors should address this as a limitation affecting the instrument's content validity.

**7. Grammar and Repetitive Writing:**

- Title Correction: There is a grammatical error in the title. It should read "What Drives..." instead of "What Drive...".
- Redundant Paragraphs: The manuscript contains instances of highly repetitive paragraphs that disrupt readability. For example, in Sections 2.1 and 2.2, the definitions of Attitude and E-WOM are stated and then immediately repeated in the next sentence using slightly different words. A thorough proofreading is necessary to eliminate these redundancies.

Is the work clearly and accurately presented and does it cite the current literature?

Partly

If applicable, is the statistical analysis and its interpretation appropriate?

Partly

Are all the source data underlying the results available to ensure full reproducibility?

Partly

Is the study design appropriate and is the work technically sound?

Partly

Are the conclusions drawn adequately supported by the results?

Partly

Are sufficient details of methods and analysis provided to allow replication by others?

No

Reviewer Expertise:

Marketing Management, Consumer Behavior, Marketing and Sustainability, Quantitative Methods (PLS-SEM)

I confirm that I have read this submission and believe that I have an appropriate level of expertise to confirm that it is of an acceptable scientific standard, however I have significant reservations, as outlined above.

# Response

**Published:** 2026-03-25

**Authors:** Muhammad Anwar Fathoni

**Affiliations:** Islamic Economics, Universitas Pembangunan Nasional Veteran Jakarta, Jakarta, Special Capital Region of Jakarta, Indonesia

Thank you very much for your time and for the valuable comments and suggestions provided on our manuscript. We greatly appreciate your careful review, which is very helpful for improving the quality and clarity of the article.

 We will carefully consider the points raised in your review and will make every effort to accommodate the relevant comments in the revised version of the manuscript in order to further enhance its overall quality.

 Once again, we sincerely thank you for your constructive feedback and support in improving this manuscript.

# Response

**Published:** 2026-04-28

**Authors:** Muhammad Anwar Fathoni

**Affiliations:** Islamic Economics, Universitas Pembangunan Nasional Veteran Jakarta, Jakarta, Special Capital Region of Jakarta, Indonesia

We sincerely thank the reviewer for the insightful and constructive comments. We have substantially revised the manuscript by strengthening the theoretical foundation through an extended TPB framework, clarifying the conceptual contribution, and improving the methodological rigor. Specifically, we have (1) elaborated the theoretical integration of E-WOM and religiosity, (2) added proper citations for measurement instruments, (3) conducted and reported Common Method Bias testing, (4) completed the structural model evaluation including Q², PLSpredict, and SRMR, (5) corrected the sampling methodology, and (6) addressed potential item redundancy and language issues. These revisions have significantly improved the clarity, rigor, and contribution of the manuscript.
